# The association between maternal smoking during pregnancy and dental development in offspring: a systematic review

**DOI:** 10.1038/s41432-025-01168-x

**Published:** 2025-05-29

**Authors:** Harry Tiernan, Muhammad Masud, Sawyer Lange, Shanika Nanayakkara, Thilini N. Jayasinghe

**Affiliations:** 1 The University of Sydney School of Dentistry, Faculty of Medicine and Health, University of Sydney, Sydney, NSW, Australia; 2 The Charles Perkins Centre, Faculty of Medicine and Health, University of Sydney, Sydney, NSW, Australia

**Keywords:** Dental caries, Diseases

## Abstract

**Aim:**

To investigate the effect of maternal smoking during pregnancy (MSDP) on the development of dental conditions in human offspring, as presented in current literature.

**Methods:**

Observational studies from six databases (MEDLINE, Embase, CINAHL, Scopus, Web of Science and Maternity and Infant Care (MIC)) were systematically searched in July 2024. Articles were screened based on their investigation of the effect of MSDP on the development of dental conditions in offspring. Methodological quality was assessed using the Newcastle Ottawa Scale (NOS), while the quality of evidence was evaluated using the Grading of Recommendations, Assessment, Development, and Evaluations (GRADE) approach.

**Results:**

A total of 17 articles were included in this review, focusing on the primary dental developmental outcomes: molar incisor hypomineralisation (MIH), enamel defects (other than MIH), missing teeth, dental eruption, and short root anomaly. Due to high levels of heterogeneity among the studies, meta-analysis was not performed. The majority of studies demonstrated good methodological quality (*n* = 10), with three assessed as fair and four as poor. The quality of evidence was categorised, with three outcomes receiving a low quality of evidence classification, and tooth eruption and short root anomaly being classed as very low quality. Statistically significant associations between MSDP and each dental outcome had varied results across studies. Most studies concluded an association between MSDP and conditions such as enamel defects (other than MIH), missing teeth, and short root anomaly. Some studies found associations with MIH, while the majority found no link between MSDP and tooth eruption in offspring.

**Conclusion:**

This review suggests a potential association between MSDP and dental development conditions in offspring. However, due to the low quality of evidence and inconsistencies in findings across observational studies, a definitive association cannot be drawn. Further, novel and high-quality research is needed to understand the impact MSDP on dental development in offspring.

Key points

- MSDP was associated with enamel defects (other than MIH), hypodontia and short root anomaly.
- Some studies indicated a link between MSDP and MIH.
- Dental eruption patterns were not significantly associated with MSDP.
- Period of MSDP and number of cigarettes consumed were associated with conditions.
- The quality of evidence generated was of low or very low quality.

## Introduction

Conditions affecting the orofacial complex can significantly hamper the quality of life of individuals^1^. Understanding the potential causes of these conditions is critical in developing education and early intervention strategies for parents and their babies. It is accepted that in addition to genetic variations, complex combinations of environmental and maternal exposures to noxious stimuli can disrupt molecular pathways during early development stages, ultimately contributing to conditions arising from defects in tooth morpho/agenesis^2^. Maternal smoking during pregnancy (MSDP) is recognized as a human teratogen and risk factor for adverse health outcomes and developmental disorders, including those involving the craniofacial complex^3^. In 2021, one in 12 Australian women reported smoking during pregnancy^4^. This is particularly significant given that tooth development begins around the sixth week in utero and continues throughout gestation and the first years of life. Disturbances during these stages have the potential to affect tooth number, shape and mineralization^5^.

Tooth development, or odontogenesis, involves the formation of three hard tissues—enamel, dentine, and cementum—along with the neurovascular dental pulp^6^ Odontogenesis begins at around six weeks in utero and continues into the first few months after birth^7^. This process is divided into four stages, classified based on their histological appearance: initiation which begins at six weeks in utero, bud (eight weeks in utero), cap (nine weeks in utero), and bell stages (11 weeks in utero)^8^. The eruption of the primary dentition can vary but typically starts from 8.6 ± 2.0 (mean ± SD) months and continues until 27.9 ± 4.4 months^9^, while their permanent counterparts replace them between 6.0 ± 2.0 years and 12.0 ± 1.9 years^10^. The crowns of primary teeth mineralize by one year, whilst permanent teeth complete crown formation from 8 to 9 year ± 9.0 (mean ± SD) months (excluding third molars)^11,12^. Root formation continues two to three years after tooth eruption^12^.

Disruptions during these critical windows of tooth development and enamel formation often can only be detected later through chemical, physical and radiographic examination. Impacting processes within these stages may have the potential to influence the number or morphological characteristics of developing teeth. For instance, an interruption during the initiation phase may affect dental placode formation thus affecting tooth number. Likewise, key morphological features of the tooth, such as cusp position and crown size, are determined in the bell stage and thus could be affected if disrupted^13^. Alterations to the normal development of the tooth may also manifest as enamel hypomineralisation or irregular tooth eruptions, both of which significantly impact on the health of the individual’s dentition and quality of life^14^.

Despite a general understanding of how tooth development progresses, there are gaps in research that directly connect MSDP to specific dental anomalies. While conditions like caries^15–17^ and cleft lip/palate^18,19^ are reported to be associated with MSDP, other dental abnormalities such as hypodontia, irregular tooth eruption, short roots, molar incisor hypomineralisation (MIH), and other enamel defects remain underexplored. Hypodontia is the failure of development of six teeth or less^20^. Irregular tooth eruption describes an eruption pattern that differs to the statistical average population data collected by previous literature for deciduous and permanent dentition^21,22^. Short root anomaly is classed when the root-crown ratio is ≤1.0^23^. MIH and enamel defects are qualitative defects causing hypomineralisation of enamel on the surface^24,25^. These anomalies can significantly affect a child’s oral function and aesthetics, with potential long-term impacts on self-confidence and quality of life^1^. Given that these conditions can arise from disruptions to the tooth development process in utero, MSDP may have an association with their occurrence.

MSDP has long been recognized as a public health issue, linked to adverse outcomes such as low birthweight, preterm birth, miscarriage, and ectopic pregnancies^26^. As recognized earlier, disruption to any of the numerous phases of odontogenesis has the potential to result in abnormalities in the dentition. Research directly examining MSDP as a primary factor influencing dental abnormalities has been limited and inconsistent. Given these uncertainties, this review aims to comprehensively assess current literature to determine whether MSDP has a direct effect on the development of teeth, focusing on anomalies such as deviations in root and crown shape, tooth number, hard tissue mineralization, and eruption patterns.

## Materials and methods

This systematic review was conducted following the Preferred Reporting Items for Systematic Reviews and Meta-Analyses (PRISMA) guidelines^27–29^.

### Developing a focused question

Using the PICO format, a focused research question was developed to include the population of human offspring, intervention group of pregnancies where the mother smoked during, comparator being pregnancies where the mother did not smoke and our outcome being the presence of dental developmental conditions. The research question for this systematic review was “What is the effect of smoking during pregnancy on the occurrence of dental development conditions in offspring when compared to non-smoking controls?”.

### Selection criteria

This review included observational studies such as cross-sectional, longitudinal/cohort studies and case-control studies performed on human participants. Studies published from 2000 – July 2024, written in the English language and have the full text available were included. Assessing a dental developmental outcome and including participant groups where the mother smoked during pregnancy and those that did not were also considered as inclusion criteria. The age of inclusion for the offspring in studies was from 0 to 19 years. Articles that were reviews or editorials, did not include a dental development outcome or were not assessing the outcome in offspring of mothers who either smoked or did not during pregnancy were excluded.

### Search strategy

A search strategy was developed to cover three main parameters of our PICO question: smoking, mothers/pregnant women, and dental developmental conditions. In total, six electronic databases were searched, MEDLINE, Embase, CINAHL, Scopus, Web of Science and Maternity and Infant Care (MIC) using the search strategy outlined in Appendix 1. Grey literature and articles from bibliographies were also screened for inclusion.

### Data extraction and synthesis

Articles captured in the database search were exported into Covidence. After removing the duplicates, title and abstract screening was completed by two independent reviewers (HT and MM) with any discrepancies resolved through discussion and where necessary with the help of a third reviewer (SL). Articles shortlisted in title and abstract screening progressed to full-text review using the above listed inclusion and exclusion criteria by two independent reviewers (HT and SL). Any disagreements between the two reviewers were solved through discussion or with the assistance of a third reviewer (MM). Data from selected articles was extracted by two independent reviewers (HT and MM) with any discrepancies resolved through a third reviewer (SL), into a predesigned extraction file using Microsoft Excel (Version 16.88). Extracted data included study design, population characteristics, outcomes measured and statistical results including study estimates, confidence intervals and *p* values. Extracted data was assessed for suitability to conduct meta-analysis, however, due to the perceived high heterogeneity of the primary studies, this was not completed. Sources of heterogeneity from included studies involved the differences in study designs, variability in each studies definition of maternal smoking as the exposure and differences between how individual outcomes were measured across studies. Additionally, individual studies differed in their adjustment for confounders further limiting the ability for meta-analysis to be performed.

### Quality assessment

To assess the methodological quality of each article, the Newcastle Ottawa Scale (NOS) was used^30^. Assessment of both case-control and cohort studies was completed using a selection score from 0 to 4, comparability score from 0 to 2 and an outcome score from 0 to 3^30^. Assessment of methodological quality in cross-sectional studies was conducted using an adapted form of the NOS to best reflect these studies^31^. As such, selection criteria could score from 0 to 3, comparability from 0 to 2 and outcomes from 0 to 2. All three reviewers (HT, HM, SL) completed quality assessment so that each article was assessed by two independent reviewers with the third to assist in solving any discrepancies.

A final quality score was assigned to each article to correlate with the quality of methodology. Good quality was defined as three or four points in selection and two points in comparability and two or three points in outcome/exposure domain. Fair quality included two points in selection and one or two points in comparability and two or three points in outcome/exposure. Finally, Poor quality consisted of zero or one point in selection or zero points in comparability or zero to one point in outcome/exposure domain (Reproduced from^18^).

An assessment of the quality of evidence for each identified dental developmental outcome was assessed using the Grading of Recommendations, Assessment, Development, and Evaluations (GRADE) framework^32^. Outcomes were assessed independently by three reviewers (HT, MM and SL), any discrepancies were resolved through discussion. Quality of evidence for the included studies was assessed initially based on the study type and then adjusted based on the performance in outcomes of methodological limitations, indirectness, imprecision, inconsistency and publication bias. Scores for each category were graded as either not serious, borderline or serious. The combined results of these were then used to give an overall rating to the evidence quality using the categories high, moderate, low or very low.

## Results

### Study selection

**Fig. 1:**
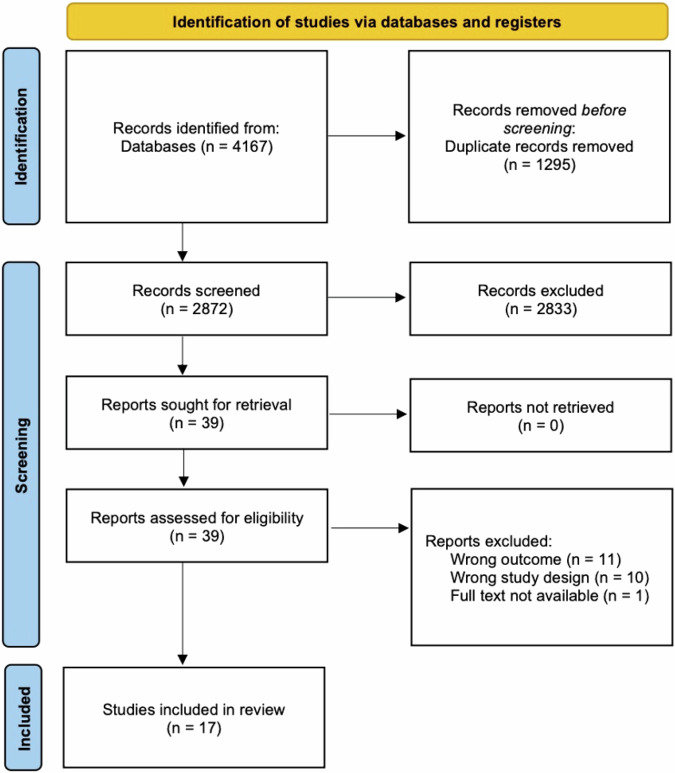
PRISMA 2020 flow diagram of the study selection process. The figure outlines the number of records identified, screened, excluded, and included at each stage of the review, following the PRISMA 2020 guidelines.

Figure 1 presents the review process in a PRISMA flowchart. From the six databases searched, 4167 articles were identified, no additional studies were identified through manual or grey literature searches. Following duplicate removal, 2872 citations had their title and abstract screened with 39 progressing to full-text review. Of the articles screened during full-text, 22 were excluded for the following reasons: 11 for assessing the wrong outcome, 10 for using the wrong study design, and one due to the full-text not being available (Appendix 2). As such, 17 articles were included in the final review presented.

### Characteristics of studies

The characteristics of included studies is summarised in Table 1. Studies were grouped based on the outcome assessed. Of the included studies, six evaluate the effect of MSDP on MIH, three assessed enamel defects other than MIH, two investigated missing teeth, five eruption timing, and one investigated short root anomaly. Categorising MIH studies separate to other enamel defect studies aimed to facilitate accurate comparisons between these specific outcomes. Studies grouped under enamel defects other than MIH included examination of hypomineralised second primary molars (HSPM), enamel hypoplasia and enamel opacities. Articles were published from 2007 until 2023. In total, there were four case-control, three cross-sectional and ten cohort studies. The number of participants was defined as the number of offsprings who were assessed for the dental development outcomes which varied from 102 to 5536. The age of participants was defined as the age recorded of the offspring participating in the study which ranged from six months to 19 years. Of the included studies, most examined smoking at any stage of pregnancy, two studies examined smoking during specific trimester of pregnancy and were noted. The dose of cigarette smoking was only investigated in two studies whilst most simply examined any amount of smoking.

**Table 1:** Characteristics of included studies.

Author, Year, Country	Study design	Participants (*n*)^a^	Age of offsprings (yrs)	Period of smoking during pregnancy	Effect of smoking dose^b^
**Molar Incisor Hypomineralisation**					
Bagattoni et al.^36^ Italy	Case-control	140 (70 cases, 70 controls)	5–17	Any	No
Lee et al.^37^ South Korea	Case-control	1191 (607 case, 584 controls)	8–11	Any	No
Lim et al.^38^ United Kingdom	Prospective Cohort	5536	7–10	Any	No
Boyer et al.^34^ France	Prospective Cohort	498	12	At conception	No
Franco et al., 2023, Brazil	Prospective Cohort	590	18–19	Any	No
Souza et al.^40^ Brazil	Retrospective Cohort	903	6–12	Any	No
**Enamel defects (other than MIH)**					
Silva et al.^33^ Australia	Prospective Cohort	172	6.78	T1, T2, T3, any^c^	No
Ford et al.^42^ Australia	Case-control	313 (104 EH cases, 104 EO cases, 105 controls)	10.2–12.1 (EH)	Any	No
			10.5–12.4 (EO)		
			10.7–12.6 (Control)		
Velló et al.^41^ Spain	Cross-sectional	102	4–5	Any	No
**Missing teeth**					
Al-Ani et al.^43^ New Zealand	Case-control	89 (case) 253 (control)	15.9 (case) 16.9 (control)	Any	Yes (1–9/day, >10/day)
Kang et al.^44^ Japan	Prospective Cohort	772	12–15	Any	Yes (1–5/day)
**Eruption**					
Bastos et al.^45^ Brazil	Prospective Cohort	359	5–6	Any	No
Ntani et al.^21^ United Kingdom	Prospective Cohort	2,915	1–2	Any	No
Ządzińska et al., 2016, Poland	Prospective Cohort	480	9–54 months	Any	No
Aktoren et al.^22^ Turkey	Retrospective Cohort	176	6–36 months	T1^c^	No
Hanioka et al.^35^ Japan	Cross-sectional	388	18–23 months	Every day or sometimes/ceased partway	No
**Short root anomaly**					
Sagawa et al.^23^ Mongolia	Cross-sectional	558	8–16	Any	No

*MIH* molar incisor hypomineralisation, *EH* enamel hypoplasia, *EO* enamel opacity.

^a^Number of offsprings recruited and assessed for dental development outcome.

^b^‘No’ indicates studies reporting smoking only as a binary variable without smoking dose.

^c^T1, First trimester; T2, Second trimester; T3, Third trimester; and ‘Any’ refers to any period during the pregnancy.

Of the included studies, 13 defined smoking during pregnancy as any period, whilst Silva et al.^33^ examined it at each trimester. Boyer et al.^34^ recorded information on smoking at the beginning of the study, Aktoren et al.^22^ only recorded smoking during the first trimester of gestation and Hanioka et al.^35^ categorised smoking as occurring every day or sometimes/ceased partway. Finally, 15 of the 17 studies recorded smoking as either a binary yes or no, whilst only two studies reported the volume of cigarette consumption during the pregnancy.

### Confounders

The confounders for each included study as listed by the authors is evident in Table 2. Of the included studies, seven did not mention any adjustments for confounders in their analysis. The remaining studies adjusted for a variety of confounders including sex, age, socioeconomic status and some pre and peri-natal maternal factors.

**Table 2:** Confounders adjusted for in included studies.

Author, Year, Country	Confounders
**Molar Incisor Hypomineralisation**	
Bagattoni et al.^36^ Italy	Not mentioned
Lee et al.^37^ South Korea	Sex, age, maternal stress during pregnancy, health supplement intake (prenatal), respiratory infection during the first three years of life, and outdoor activity time for children
Lim et al.^38^ United Kingdom	Age, education, parity (maternal), alcohol consumption
Boyer et al.^34^ France	Not mentioned
Franco et al., 2023, Brazil	Not mentioned
Souza et al.^40^ Brazil	Not mentioned
**Enamel defects (other than MIH)**	
Silva et al.^33^ Australia	Age, socioeconomic indexes, vitamin D level at birth, cord attachment, chorionicity
Ford et al.^42^ Australia	Not mentioned
Velló et al.^41^ Spain	Not mentioned
**Missing teeth**	
Al-Ani et al.^43^ New Zealand	Maternal age at delivery, female sex, gestational age of child, household socio-economic background,
Kang et al.^44^ Japan	Sex, gestational age, maternal BMI, maternal age at delivery, maternal education, alcohol consumption during pregnancy, paternal smoking
**Eruption**	
Bastos et al.^45^ Brazil	Age
Ntani et al.^21^ United Kingdom	Age, sex
Ządzińska et al., 2016, Poland	Sex, gestational age
Aktoren et al.^22^ Turkey	Not mentioned
Hanioka et al.^35^ Japan	Gender, location of residence, birth order, parent’s education level, annual income
**Short root anomaly**	
Sagawa et al.^23^ Mongolia	Sex, gestational age, family income, maternal education, maternal age at delivery

#### Molar incisor hypomineralisation

The outcomes of the six studies investigating MIH all compared the presence of this defect in offspring and MSDP (Table 3). The results varied, with an equal number of studies demonstrating a significant association or an increased incidence of MIH and MSDP as those showing no association. Two studies reported a statistically significant difference in the presence of MIH in offspring between the mothers who smoked during pregnancy and those who did not^36,37^. An increased incidence of MIH in offspring exposed to MSDP was also found (OR = 2, 95% CI 0.8–4.4)^34^. In contrast, Lim et al. found that, after adjusting to confounders, the incidence of MIH in offspring of smoking mothers attenuated to the null (OR = 0.98, 95% CI 0.608–1.38)^38^. Franco et al. found no significant association between MIH and MSDP in molars alone and molars and incisors measured together (*p *= 0.15 and *p *= 0.58, respectively)^39^. In addition, a retrospective study concluded that there was no significant association between smoking status during pregnancy and MIH in children born in both urban and rural settings (*p *= 0.602 and *p *= 0.853, respectively)^40^.

**Table 3:** Summary of results of included studies on the association between MSDP and dental anomalies.

Author, Year, Country	Dental anomaly	Exposure (MSDP period)	*n* yes smoking/total participants^a^	Study estimate (Odds ratio/relative risk)	CI (95%)	*p* value
**Molar incisor hypomineralisation**						
Bagattoni et al.^36^ Italy	MIH - Cases	Anytime	6/70	0.93	0.24–3.66	**0.0005**
	MIH - Controls	—	25/70			
Lee et al.^37^ South Korea	MIH – Cases	Anytime	40/607	2.37	1.15–4.88	**0.012**
	MIH – Cases	Anytime	40/562	2.37^b^	1.15–4.88	**0.019**
	MIH – Controls	Anytime	20/584			
	MIH – Controls	Anytime	20/560^b^			
Lim et al.^38^ United Kingdom	MIH	Anytime	73/1072	1.41	1.06–1.86	—
	MIH	Anytime		0.98^b^	0.68–1.38	—
Boyer et al.^34^ France	MIH	At conception	6/47	2	0.8–4.4	—
Franco et al., 2023, Brazil	MIH (molars)	Anytime	28/590	—	—	0.15
	MIH (molars or incisors)	Anytime		—	—	0.58
Souza et al.^40^ Brazil	MIH (urban)		25/108	1.14	0.69–1.87	0.602
	MIH (rural)		15/74	1.06	0.55–2.05	0.853
**Enamel defects (other than MIH)**						
Silva et al.^33^ Australia	HSPM	Anytime	88/344	1.52	0.77–3.00	0.225
	HSPM	T1	86/344	1.58	0.80–3.13	0.185
	HSPM	T2	40/344	2.84	1.28–6.30	**0.01**
	HSPM	T3	40/344	3.23	1.42–7.33	**0.005**
	HSPM	T2/T3	—	2.28^b^	0.98–14.30	0.055
	HSPM	T2/T3	—	3.85^b^	1.44–10.29	**0.007**
Ford et al.^42^ Australia	Enamel hypoplasia in permanent dentition	Anytime	19/104	3.4^c^	1.3–8.9	**0.007**
Velló et al.^41^ Spain	Enamel opacity	Anytime	—	—	—	0.274
	Enamel hypoplasia	Anytime		—	—	**0.028**
	Combined effects (opacity and hypoplasia)	Anytime		—	—	0.246
**Missing teeth**						
Al-Ani et al.^43^ New Zealand	Cases					
	Missing teeth	Anytime	18/89	—	—	**0.004**
	Missing teeth and smoking 1-9/day	Anytime	9/89	2.05	0.73–5.75	0.232
	Missing teeth and smoking <10/day	Anytime	9/89	4.18	1.49–11.80	**0.007**
	Controls					
	Smoking (any)	Anytime	22/253	—	—	—
	Smoking (1–9)	Anytime	13/253	—	—	—
	Smoking (< 10)	Anytime	9/253	—	—	—
Kang et al.^44^ Japan	Missing teeth and smoking 1-5/day	Anytime	—	2.89	0.63–13.19	—
	Missing teeth and smoking 1–5/day	Anytime	17/772	2.80^b^	0.52–15.06	—
	Missing teeth and smoking 6 + /day	Anytime	—	3.47	1.14–10.56	**0.014**
	Missing teeth and smoking 6 + /day	Anytime	29/772	4.59^b^	1.07–19.67	**0.024**
**Eruption**						
Bastos et al.^45^ Brazil	*n* pairs of teeth emerged at 6 months	Anytime	119/359	—	0.6–1.6	0.978
	*n* pairs of teeth emerged at 12 months	Anytime		—	0.9–1.1	0.659
	26 emergence 6 y	Anytime		—	0.8–1.2	0.904
	46 emergence 6 y	Anytime		—	0.8–1.2	0.785
Ntani et al.^21^ United Kingdom	Age of first tooth eruption	Anytime	—	1.122^c^	1.01–1.25	**0.035**
	Number of teeth at 1 y	Anytime	—	1.113^c^	1.06–1.17	**<0.001**
	Advanced dental development at 2 y	Anytime	—	1.283^c^	1.10–1.50	**0.002**
Ządzińska et al., 2016, Poland	Variation in age of first tooth eruption	Anytime	243/480	—	—	**0.0064**
Aktoren et al.^22^ Turkey	Early stage eruption	Anytime	5/26	1.66	0.54–5.13	0.561
	Normal stage eruption	Anytime	14/112	—	—	—
	Late stage eruption	Anytime	3/38	0.6	0.16–2.21	0.632
Hanioka et al.^35^ Japan	Medium dose with ETE	Anytime	32/257	1.12	0.58–2.16	0.732
	Medium dose with ETE	Anytime		0.95^b^	0.45–1.98	0.888
	High dose with ETE	Anytime	10/257	1.75	0.47–6.49	0.402
	High dose with ETE	Anytime		1.42^b^	0.34–5.96	0.631
**Short root anomaly**						
Sagawa et al.^23^ Mongolia	Short root anomaly	Anytime	7/79	4.68	1.73–12.72	**0.002**
	Short root anomaly	Anytime	7/79	4.95^b^	1.65–14.79	**0.004**
	No short root anomaly	Anytime	10/479			
	No short root anomaly	Anytime	10/479^b^			

*MIH* molar incisor hypomineralisation, *HSPM* hypomineralised second primary molars, *MSDP* maternal smoking during pregnancy, *ETE* early tooth eruption, *y* year, *n* number.

Bold values are statistically significant.

^a^The number of mothers who responded yes to smoking during pregnancy as a proportion of the total study population.

^b^Where results were adjusted for confounders. Where no symbol is evident, unadjusted values are displayed.

^c^Where relative risk was used as the study estimate unit over odds ratio.

#### Enamel defects other than MIH

Across the three included studies, all demonstrated an association between MSDP and enamel defects that were not MIH, specifically enamel hypoplasia (Table 3). The study by Silva et al.^33^ which analysed data based on specific periods of smoking throughout pregnancy demonstrated that maternal smoking during the second and third trimesters were statistically significant (*p *= 0.01 and 0.005, respectively)^33^. However, their analysis based on smoking at any time during pregnancy, as well as smoking limited to the first trimester, was not statistically significant (*p *= 0.225 and *p *= 0.185, respectively)^33^. They also combined the second and third trimester and analysed using two models (with and without the covariate of birth vitamin D data), the first model showed no statistical significance (*p *= 0.055), whilst the second model indicated a significant association (*p *= 0.007)^33^. Vello et al. examined both enamel hypoplasia and enamel opacity as outcomes for enamel defects^41^. While the presence of enamel opacities was not statistically significant (*p *= 0.274), enamel hypoplasia alone showed a significant association with MSDP (*p *= 0.028). However, the combined effect of both these defects was not significant (*p *= 0.246)^41^. A case control study conducted by Ford et al. also concluded that there was a significant association between MSDP and the combined enamel defects measured, compared to controls (*p *= 0.007)^42^.

#### Missing teeth (hypodontia)

The prevalence of hypodontia and MSDP was seen to be significant and associated with the number of cigarettes consumed (Table 3). A case control study reported that smoking at any level during pregnancy was significantly associated with the presence of missing teeth in the offspring (*p *= 0.004)^43^. However, when examining the dose relationship by categorising the participants as either one to nine cigarettes per day or smoking 10+ cigarettes per day, only smoking 10+ cigarettes per day was statistically significant (*p *= 0.007) with the presence of hypodontia, whilst smoking one to nine cigarettes per day was not (*p *= 0.232)^43^. Results presented by Kang et al.^44^ also found that, after adjusting for confounders, smoking 6+ cigarettes per day was statistically significantly associated with missing teeth (*p *= 0.024) even though a similar observation was not present in the participants who smoked 1-5 cigarettes per day. However, the authors noted an odds ratio greater than 1 for this group (OR = 2.8, 95% CI: 0.52-15.06)^44^.

#### Eruption

The investigation of dental eruption patterns provided varied results (Table 3). Whilst some studies found an association between MSDP and altered dental eruption, the majority did not. A prospective cohort study conducted in Brazil showed that there was no statistically significant association between MSDP and any observed tooth emergence patterns in offspring and MSDP^45^. Similarly, Hanioka et al. found no significant association between high or medium doses of smoking and early tooth eruption, both in crude results and after adjusting for confounders (*p *= 0.888, *p *= 0.631)^35^. Another retrospective cohort study also reported no statistically significant association between MSDP and either early or late-stage eruption (*p *= 0.561, *p *= 0.632)^22^. However, Ntani et al. reported statistically significant associations, reporting that MSDP was associated with early eruption of the first tooth (*p *= 0.035), a greater number of teeth present at one year old (*p *= <0.001), and a higher likelihood of having more than 16 teeth by the age of two (*p *= 0.002) in children whose mothers smoked during pregnancy^21^.

#### Short root anomaly

Only one study investigated the effect of MSDP on root development and identified a significant association with MDSP (Table 3). This study reported both crude and adjusted results, indicating that MSDP was significantly associated with short root anomaly (*p *= 0.002, *p *= 0.004, respectively)^23^. The adjusted odds ratio showed that offspring of mothers who smoked were more likely to develop short root anomaly compared to non-smokers (OR = 4.95, 95% CI: 1.65–14.79)^23^.

### Quality assessment of included studies

#### Newcastle-Ottawa Scale (NOS)

The studies included were categorsied based on their study design using the NOS, as shown in Table 4. Of these studies, ten were rated as good quality, three as fair quality, and four as poor quality. The studies rated as poor quality failed to meet the required standards either in the comparability of subjects or in the measurement of study outcomes.

**Table 4:** Quality assessment using the Newcastle Ottawa Scale (NOS).

Author	Year	Study design	Selection	Comparability	Outcomes	Quality
Lee et al.	2020	Case-control	****	**	**	Good
Ford et al.	2009	Case-control	****	**	***	Good
Al-Ani et al.	2017	Case-control	****	**	***	Good
Bagattoni et al.	2022	Case-control	****	**	***	Good
Nakagawa Kang et al.	2019	Cohort	***	**	***	Good
Franco et al.	2023	Cohort	***	**	**	Good
Bastos et al.	2007	Cohort	**	**	**	Fair
Boyer et al.	2023	Cohort	***		***	Poor
Souza et al.	2012	Cohort	***	**	***	Good
Silva et al.	2019	Cohort	***	**	**	Good
Ntani et al.	2015	Cohort	***	**	***	Good
Ządzińska et al.	2016	Cohort	***	**	*	Poor
Aktoren et al.	2010	Cohort	****		**	Poor
Lim et al.	2022	Cohort	***	*	*	Poor
Hanioka et al.	2018	Cross-sectional	**	**	**	Fair
Sagawa et al.	2021	Cross-sectional	**	**	**	Fair
Velló et al.	2010	Cross-sectional	***	**	**	Good

Good quality: 3 or 4 stars (*) in selection domain AND 2 stars in comparability domain AND 2 or 3 stars in outcome/exposure domain. Fair quality: 2 stars in selection domain AND 1 or 2 stars in comparability domain AND 2 or 3 stars in outcome/exposure domain. Poor quality: 0 or 1 star in selection domain OR 0 stars in comparability domain OR 0 or 1 stars in outcome/exposure domain (Reproduced from Ref.^18^).

#### GRADE scale

The overall quality of evidence for each dental development outcome is summarised in Table 5. The GRADE scale analysis indicated that the evidence related to MIH, enamel defects, and missing teeth was of low quality, whilst studies examining eruption patterns and short root anomaly were classified as very low quality. Overall, the studies presented showed a lack of high quality evidence based on methodological limitations, indirectness, imprecision, inconsistency and publication bias.

**Table 5:** Quality of evidence assessed using GRADE framework.

Outcome	Methodological limitations	Indirectness	Imprecision	Inconsistency	Publication Bias	Final rating
MIH	Borderline	Not serious	Borderline	Serious	Not suspected	Low
Enamel defects (other than MIH)	Borderline	Not serious	Borderline	Borderline	Not suspected	Low
Missing teeth	Not serious	Not serious	Borderline	Not serious	Not suspected	Low
Eruption	Serious	Not serious	Not serious	Serious	Not suspected	Very Low
Short root anomaly	Borderline	Not serious	Serious	–	Not suspected	Very Low

*MIH* molar incisor hypomineralisation.

Results were assessed for all studies identified across each dental development outcome. Quality was assessed as being either Serious, Borderline or Non-serious. Where an outcome could not be completed due to only having a single study to compare the column was marked with a dash. The final rating for each outcome was determined as either being High, Moderate, Low or Very Low quality.

## Discussion

Dental developmental conditions describe anomalies that may affect the number, shape, size, or colour of teeth^46^ and can significantly impact quality of life^1^. With MSDP already being linked to conditions such as caries^15^ and cleft-lip/palate^18^, along with Australia’s high prevalence of maternal smoking^4^, investigation into the effect of MSDP on tooth development is necessary. This review aimed to investigate the effect of MSDP on dental developmental conditions affecting tooth shape, size, number, roots and hard tissue development. From the literature reviewed, associations between MSDP and enamel defects and hypodontia were identified, however quality of the evidence was generally low, with some studies indicating a link with MIH as well.

Current evidence suggests a plausible mechanism between MSDP and dental anomalies involves increased oxidative stress^47,48^ and placental hypoxia^49^. Both passive and active smoking during pregnancy can adversely affect the neural crest cells responsible for forming deciduous teeth, potentially preventing their maturation and resulting in hypodontia^47,48^. Chronic foetal hypoxia due to nicotine exposure may lead to developmental delays, further inhibiting proper tooth development^13,50^. Additionally, nicotine can disrupt the deposition of enamel and dentine matrices, leading to inadequate mineralisation and resulting in hypomineralisation and enamel defects^51^.

Several studies have demonstrated a positive association between MSDP and MIH^34,36,37^. These results are in contrast to a recent review examining the aetiology of MIH, which, following a meta-analysis, reported no significant association between MSDP and MIH^52^. Notably, four out of the six MIH studies included in this review were published after the systematic review examining the aetiology of MIH by Garot et al., with three of these findings contributing to the observed significant positive associations^36,37^ or an increased incidence of MIH^34^. This discrepancy may stem from the inclusion of more recent studies in our review. In addition to MIH, other enamel defects were also positively associated with MSDP^33,41,42^. With both conditions involving the defects in the development of enamel it is unsurprising that a correlation exists. Specifically, significant association between enamel hypoplasia and MSDP were found^41^, particularly with maternal smoking during the second or third trimesters^33^. Animal studies have further demonstrated that smoking negatively impacts ameloblast function, which is the cell type responsible for enamel development^53^. These mechanisms highlight the potential adverse effects of nicotine and smoking on enamel formation, supporting the associations identified between MSDP, enamel defects and possibly MIH.

An association between hypodontia and MSDP is also supported by the literature^43,44^. Both a New Zealand case-control study^43^ and a Japanese cohort study^44^ indicate a dose-dependent relationship between hypodontia and MSDP. Specifically, Al-Ani et al. identified that smoking ten or more cigarettes per day increases the risk of hypodontia^43^, whilst Nakagawa Kang et al. reported a similar association but with six or more cigarettes^44^. The presence of a dose-dependent relationship strengthens the evidence of a causal relationship between MSDP and hypodontia rather than a mere association. Identification of this biological gradient aligns with the parameters from Bradford Hills criteria for causation^54^. This relationship suggest that elevated levels of smoking-related toxins may heighten the risk of hypodontia, potentially through increased oxidative stress damaging neural crest cells^13,47,48,50^.

Most studies did not find a significant association between MSDP and tooth eruption patterns. The aetiology of dental eruption patterns is likely to be a complex interconnected process between both environment and genes^55^. A recent review indicates that the Runt-related transcription factor-2 (RUNX2) gene plays a key role in dental eruption^56^, with delays in eruption previously linked to malnutrition and low birthweight^57^. It has been hypothesised that smoking during the first trimester which coincides with the initiation, bud and cap stages of odontogenesis, may lead to accelerated eruption timings^51^. Despite most studies showing no association, two studies reported a significant association^21,58^, consistent with previous research^59^. The overall evidence for this relationship was rated very low, largely due to the reliance on self-reported data from mothers regarding eruption timing and a lack of consensus on measuring maternal smoking, which may have contributed to conflicting results. Only a single study examining short root anomalies was identified, which showed an association with MSDP, however, the lack of comparability among studies rendered the evidence very low quality. Smoking has been shown to induce DNA methylation of the EVC2 protein complex^60^, which mediates epithelial-mesenchymal interactions that influence root length^61,62^. While some evidence suggests an association a possible association between MSDP and short roots, the lack of studies limits clinical applicability.

A key strength of this review is that it incorporates a range of different dental developmental conditions. The comprehensive search strategy used across databases allowed for an extensive search of the research and inclusion of a wide range of studies that fit the inclusion criteria. However, a meta-analysis was not conducted due to the heterogeneity in the study designs, differences in exposure and outcome definitions, outcome assessments, and analyses to adjust for the confounders. Instead, the quality of evidence was graded using a subjective measure. The studies included were graded as having either low or very low quality of evidence, an additional limitation in clinical applicability of these results. Factors significant in the poor methodological quality of studies included the risk of recall and social desirability bias. Studies relied on the self-reporting of both smoking data and in some cases the dental outcome itself. Additionally, many studies did not adjust data for key confounders including passive smoking, maternal health status or other pre and peri-natal factors such as alcohol consumption. Inconsistency in methodologies and poor comparability between studies is a significant factor in improving the clinical relevance of these results. An additional limitation of this review includes the lack of inter-rater reliability measures for assessment of agreement between reviewers during study selection and quality assessment. Further, the lack of sub-group analysis is also a limitation of this review. Sub-group analysis was not feasible due to the heterogeneity across the included studies. Specifically, there were inconsistencies in adjusting for confounders, insufficient details to allow for stratification of data by key factors including age, disease severity and exposure measurements. The incorporation of subgroup analysis could have provided further insights into the differences in effect sizes across different contexts and strengthened the rationale behind the narrative synthesis. As mentioned earlier, disruptions in tooth development at different stages can influence the type of dental anomalies in offspring^8^. However, there was insufficient evidence to draw conclusions on this aspect as only one included study examined the effects of smoking during different stages of pregnancy. Therefore, the lack of reporting of these time periods is a significant limitation of the included studies.

The importance of identifying risk factors for the development of dental conditions is critical to shaping clinical care. However, without the development of a universal measure of determining MSDP, the clinical applicability of results presented in this review is limited. Future research should focus on improving the methodological inconsistencies of current study designs to provide more high quality evidence. Reporting the specific periods of maternal smoking and dose-dependent relationships will improve the understanding on the association between MSDP and dental anomalies in offspring. Additionally, prospective cohort study designs in replacement of retrospective data collection will reduce the potential bias and improves the quality of evidence. Improvements to the measurement of maternal smoking with biomarker validation instead of reliance on self-reported data may also increase the quality of evidence and clinical applicability of these results. In addition to standardised measures of MSDP, the standardisation of the dental anomaly measurements will reduce heterogeneity and improve feasibility of meta-analyses in future. Further, adjustments for confounders such as passive smoking exposure and other environmental pollutants, genetic predisposition to anomalies, and maternal pre and peri-natal factors should be implemented to increase quality of evidence generated by future research.

In conclusion, this systematic review demonstrates that prenatal exposure to smoking does pose significant risks in the development of hypodontia and enamel defects in offspring. However, the strength of this evidence remains limited due to the methodological inconsistencies across studies. To ensure conclusive and accurate clinical interpretation of these results, further high-quality and well-controlled research is necessary to validate the risk of MSDP on development of dental conditions.

## Supplementary information

Supplementary Information

PRISMA Checklist

## Data Availability

The data supporting this article can be made available by the corresponding author upon request.
