# Supplementary material for: The association between maternal smoking during pregnancy and dental development in offspring: a systematic review
Source: Evid Based Dent. 2025 May 29;26(3):157. doi: 10.1038/s41432-025-01168-x (PMC12474549; doi:10.1038/s41432-025-01168-x)
Supplement: Supplementary file 1 — Supplementary Information [file 41432_2025_1168_MOESM1_ESM.docx]

# Appendices

**Appendix 1**

**Search strategy for MEDLINE and Embase**

((smok*/ or smok*.mp. or tobacc?o/ or tobacc?o.mp. or cigar*/ or cigar*.mp. or nicotine?/ or nicotine?.mp. or vap*/ or vap*.mp. or e-?cigar*/ or e-?cigar*.mp. or electronic smok*/ or electronic smok*.mp. or e-?smok*/ or e-?smok*.mp.) AND (pregnan*/ or pregnan*.mp. or expectan* mother*/ or expectan* mother*.mp. or child-?bear*/ or child-?bear*.mp. or gestation*/ or gestation*.mp. or gravid*/ or gravid*.mp. or matern*/ or matern*.mp. or antenat*/ or antenat*.mp. or ante-?nat*/ or ante-?nat*.mp. or prenat*/ or prenat*.mp. or pre-?nat*/ or pre-?nat*.mp. or mother*/ or mother*.mp. or fo?etus/ or fo?etus.mp. or fo?etal/ or fo?etal.mp. or bab*/ or bab*.mp. or child*/ or child*.mp. or infan*/ or infan*.mp.) AND (t##th develop*/ or t##th develop*.mp. or dent* develop*/ or dent* develop*.mp. or enamel develop*/ or enamel develop*.mp. or hypodontia/ or hypodontia.mp. or hyperdontia/ or hyperdontia.mp. or anodontia/ or anodontia.mp. or agenesis/ or agenesis.mp. or m#crodontia/ or m#crodontia.mp. or dent* defect*/ or dent* defect*.mp. or enamel defect*/ or enamel defect*.mp. or taurodont*/ or taurodont*.mp. or oligodont*/ or oligodont*.mp. or ectoderm* dysplas*/ or ectoderm* dysplas*.mp. or amelogenesis imperfecta/ or amelogenesis imperfecta.mp. or dentinogenesis imperfecta/ or dentinogenesis imperfecta.mp. or hypomineralization/ or hypomineralization.mp. or hypo-?mineralization/ or hypo-?mineralization.mp. or hypomineralisation/ or hypomineralisation.mp. or hypo-?mineralisation/ or hypo-?mineralisation.mp. or hypermineralization/ or hypermineralization.mp. or hyper-?mineralization/ or hyper-?mineralization.mp. or hypermineralisation/ or hypermineralisation.mp. or hyper-?mineralisation/ or hyper-?mineralisation.mp. or gemination/ or gemination.mp. or concrescence/ or concrescence.mp. or crown siz*/ or crown siz*.mp. or crown shap*/ or crown shap*.mp. or missing t##th/ or missing t##th.mp. or dent* disease*/ or dent* disease*.mp. or t##th disease*/ or t##th disease*.mp. or dent* care/ or dent* care.mp. or tooth germ/ or tooth germ.mp. or odontogenesis/ or odontogenesis.mp. or t##th abnormalit*/ or t##th abnormalit*.mp. or supernumerary/ or supernumerary.mp. or fused t##th/ or fused t##th.mp. or dens in dente/ or dens in dente.mp. or odontometr*/ or odontometr*.mp. or t##th crown*/ or t##th crown*.mp. or t##th root*/ or t##th root*.mp. or root distort*/ or root distort*.mp. or t##th distort*/ or t##th distort*.mp. or root defect*/ or root defect*.mp. or peg shape*/ or peg shape*.mp. or cusp* defect*/ or cusp* defect*.mp. or pulp* disease*/ or pulp* disease*.mp. or pulp* defect*/ or pulp* defect*.mp. or pulp* distort*/ or pulp* distort*.mp.))

**Search strategy for CINAHL and MIC**

("( smok* OR smok*.mp OR tobacc?o OR tobacc?o.mp OR cigar* OR cigar*.mp OR nicotine? OR nicotine?.mp OR vap* OR vap*.mp OR e-?cigar* OR e-?cigar*.mp OR electronic smok* OR electronic smok*.mp OR e-?smok* OR e-?smok*.mp ) AND ( pregnan* OR pregnan*.mp OR expectan* mother* OR expectan* mother*.mp OR child-?bear* OR child-?bear*.mp OR gestation*OR gestation*.mp OR gravid* OR gravid*.mp OR matern* OR matern*.mp OR antenat* OR antenat*.mp OR ante-?nat* OR ante-?nat*.mp OR prenat* OR prenat*.mp OR pre-?nat* OR pre-?nat*.mp OR mother* OR mother*.mp OR fo?etus OR fo?etus.mp OR fo?etal OR fo?etal.mp OR bab* OR bab*.mp OR child* OR child*.mp OR infan* OR infan*.mp ) AND ( t??th develop* OR t??th develop*.mp OR dent* develop* OR dent* develop*.mp OR enamel develop* OR enamel develop*.mp OR hypodontia OR hypodontia.mp OR hyperdontia OR hyperdontia.mp OR anodontia OR anodontia.mp OR agenesis OR agenesis.mp OR m?crodontia OR m?crodontia.mp OR dent* defect* OR dent* defect*.mp OR enamel defect* OR enamel defect*.mp OR taurodont* OR taurodont*.mp OR oligodont* OR oligodont*.mp OR ectoderm* dysplas* OR ectoderm* dysplas*.mp OR amelogenesis imperfecta OR amelogenesis imperfecta.mp OR dentinogenesis imperfecta OR dentinogenesis imperfecta.mp OR hypomineralization OR hypomineralization.mp OR hypo-?mineralization OR hypo-?mineralization.mp OR hypomineralisation OR hypomineralisation.mp OR hypo-?mineralisation OR hypo-?mineralisation.mp OR hypermineralization OR hypermineralization.mp OR hyper-?mineralization OR hyper-?mineralization.mp OR hypermineralisation OR hypermineralisation.mp OR hyper-?mineralisation OR hyper-?mineralisation.mp OR gemination OR gemination.mp OR concrescence OR concrescence.mp OR crown siz* OR crown siz*.mp OR crown shap* OR crown shap*.mp OR missing t??th OR missing t??th.mp OR dent* disease* OR dent* disease*.mp OR t??th disease* OR t??th disease*.mp OR dent* care OR dent* care.mp OR tooth germ OR tooth germ.mp OR odontogenesis OR odontogenesis.mp OR t??th abnORmalit* OR t??th abnORmalit*.mp OR supernumerary OR supernumerary.mp OR fused t??th OR fused t??th.mp OR dens in dente OR dens in dente.mp OR odontometr* OR odontometr*.mp OR t??th crown* OR t??th crown*.mp OR t??th root* OR t??th root*.mp OR root distORt* OR root distORt*.mp OR t??th distORt* OR t??th distORt*.mp OR root defect* OR root defect*.mp OR peg shape* OR peg shape*.mp OR cusp* defect* OR cusp* defect*.mp OR pulp* disease* OR pulp* disease*.mp OR pulp* defect* OR pulp* defect*.mp OR pulp* distORt* OR pulp* distORt*.mp )

**Search strategy for SCOPUS and Web of Science**

( TITLE-ABS-KEY ( smok* OR tobacc?o OR cigar* OR nicotine? OR vap* OR e-?cigar* OR ( electronic AND smok* ) OR e-?smok* ) AND TITLE-ABS-KEY ( pregnan* OR ( expectan* AND mother* ) OR child-?bear* OR gestation* OR gravid* OR matern* OR antenat* OR ante-?nat* OR prenat* OR pre-?nat* OR mother* OR fo?etus OR fo?etal OR bab* OR child* OR infan* ) AND TITLE-ABS-KEY ( ( t??th AND develop* ) OR ( dent* AND develop* ) OR ( enamel AND develop* ) OR hypodontia OR hyperdontia OR anodontia OR agenesis OR m?crodontia OR ( dent* AND defect* ) OR ( enamel AND defect* ) OR taurodont* OR oligodont* OR ( ectoderm* AND dysplas* ) OR ( amelogenesis AND imperfecta ) OR ( dentinogenesis AND imperfecta ) OR hypomineralization OR hypo-?mineralization OR hypomineralisation OR hypo-?mineralisation OR hypermineralization OR hyper-?mineralization OR hypermineralisation OR hyper-?mineralisation OR gemination OR concrescence OR ( crown AND siz* ) OR ( crown AND shap* ) OR ( missing AND t??th ) OR ( dent* AND disease* ) OR ( t??th AND disease* ) OR ( dent* AND care ) OR ( tooth AND germ ) OR odontogenesis OR ( t??th AND abnormalit* ) OR supernumerary OR ( fused AND t??th ) OR ( dens AND in AND dente ) OR odontometr* OR ( t??th AND crown* ) OR ( t??th AND root* ) OR ( root AND distort* ) OR ( t??th AND distort* ) OR ( root AND defect* ) OR ( peg AND shape* ) OR ( cusp* AND defect* ) OR ( pulp* AND disease* ) OR ( pulp* AND defect* ) OR ( pulp* AND distort* ) ) )

**Appendix 2**

| **Article reference** | **Reason for exclusion** |
| --- | --- |
| Luigi P. A lifelong smile: a journey that begins during pregnancy!. European journal of paediatric dentistry. 2024 Jun 3;25(2):89. | Wrong study design – is an editorial |
| Taylor GD. Molar incisor hypomineralisation. Evidence-based dentistry. 2017 Mar;18(1):15-6. | Wrong study design – is a review |
| Küchler EC, Scariot R, Kirschneck C. Craniofacial Growth and Development: Novel Insights. Frontiers in Cell and Developmental Biology. 2021 Aug 20;9:744711. | Wrong study design – is an editorial |
| Bernabé E, MacRitchie H, Longbottom C, Pitts NB, Sabbah W. Birth weight, breastfeeding, maternal smoking and caries trajectories. Journal of dental research. 2017 Feb;96(2):171-8. | Wrong outcome assessed - caries |
| Arora M, Austin C. Teeth as a biomarker of past chemical exposure. Current opinion in pediatrics. 2013 Apr 1;25(2):261-7. | Wrong study design – is a review |
| Mook-Kanamori DO, Steegers EA, Eilers PH, Raat H, Hofman A, Jaddoe VW. Risk factors and outcomes associated with first-trimester fetal growth restriction. Jama. 2010 Feb 10;303(6):527-34. | Wrong outcome assessed – foetal growth restriction |
| Zhou S, Rosenthal DG, Sherman S, Zelikoff J, Gordon T, Weitzman M. Physical, behavioral, and cognitive effects of prenatal tobacco and postnatal secondhand smoke exposure. Current problems in pediatric and adolescent health care. 2014 Sep 1;44(8):219-41. | Wrong study design – is a review |
| Shi M, Wehby GL, Murray JC. Review on genetic variants and maternal smoking in the etiology of oral clefts and other birth defects. Birth Defects Research Part C: Embryo Today: Reviews. 2008 Mar;84(1):16-29. | Wrong outcome assessed – oral clefts |
| Billings RJ, Berkowitz RJ, Watson G. Teeth. Pediatrics. 2004 Apr 1;113(Supplement_3):1120-7. | Wrong study design – is a review |
| Milnerowicz-Nabzdyk E, Bizoń A, Zimmer M. How does tobacco smoke affect fetal growth potential in the first trimester of pregnancy as measured by volume parameters of the fetus, trophoblast, and gestational sac?. Reproductive Sciences. 2017 Apr;24(4):548-59. | Wrong outcome assessed |
| Nielsen CH, Larsen A, Nielsen AL. DNA methylation alterations in response to prenatal exposure of maternal cigarette smoking: a persistent epigenetic impact on health from maternal lifestyle?. Archives of toxicology. 2016 Feb;90:231-45. | Wrong study design – is a review |
| Prabhu N, Smith N, Campbell D, Craig LC, Seaton A, Helms PJ, Devereux G, Turner SW. First trimester maternal tobacco smoking habits and fetal growth. Thorax. 2010 Mar 1;65(3):235-40. | Wrong outcome assessed – foetal growth |
| Shen CA, Guo R, Li W. Enamel defects in permanent teeth of patients with cleft lip and palate: a cross-sectional study. Journal of International Medical Research. 2019 May;47(5):2084-96. | Wrong outcome assessed – cleft lip and palate |
| Wuollet E, Laisi S, Salmela E, Ess A, Alaluusua S. Background factors of molar-incisor hypomineralization in a group of Finnish children. Acta Odontologica Scandinavica. 2014 Nov 1;72(8):963-9. | Wrong outcome assessed – does not include maternal smoking |
| Dent JP. Other journals in brief. J Prosthet Dent. 2016;115:741-8. | Wrong study design – is an editorial |
| Avsar A, Topaloglu B, Hazar-Bodrumlu E. Association of passive smoking with dental development in young children. Eur J Paediatr Dent. 2013 Sep 1;14(3):215-8. | Wrong outcome assessed |
| Gontarev SN, Tsimbalistov AV, Ryzhova IP, Trifonov BV, Kunitsina NM, Gontareva IS. Analysis of some of causes of congenital malformations of the face, jaws and teeth. Research Journal of Pharmaceutical, Biological and Chemical Sciences. 2015;6(4):1-3. | Full text not available |
| Bulani M, Shetiya SH, Agarwal D, Mathur A. Severe Early Childhood Caries, Hypoplasia-Associated Severe Early Childhood Caries and Deciduous Molar Hypomineralization amongst 3 to 6 Years Old Anganwadi Children in Pune, Maharashtra: A Cross-Sectional Study. Indian Journal of Public Health Research & Development. 2020 Apr 1;11(4). | Wrong outcome assessed |
| Nelson S, Albert JM, Geng C, Curtan S, Lang K, Miadich S, Heima M, Malik A, Ferretti G, Eggertsson H, Slayton RL. Increased enamel hypoplasia and very low birthweight infants. Journal of dental research. 2013 Sep;92(9):788-94. | Wrong outcome assessed – did not examine maternal smoking |
| Yang L, Wang H, Yang L, Zhao M, Guo Y, Bovet P, Xi B. Maternal cigarette smoking before or during pregnancy increases the risk of birth congenital anomalies: a population-based retrospective cohort study of 12 million mother-infant pairs. BMC medicine. 2022 Dec;20:1-7. | Wrong outcome assessed – did not examine dental development |
| De Coster PJ, Marks LA, Martens LC, Huysseune A. Dental agenesis: genetic and clinical perspectives. Journal of Oral Pathology & Medicine. 2009 Jan;38(1):1-7. | Wrong study design – is a review |
| Lima LJ, Ramos-Jorge ML, Soares ME. Prenatal, perinatal and postnatal events associated with hypomineralized second primary molar: a systematic review with meta-analysis. Clinical Oral Investigations. 2021 Dec;25(12):6501-16. | Wrong study design – is a review |
